# Association of Collagenous Gastritis With Helicobacter pylori Infection

**DOI:** 10.7759/cureus.42172

**Published:** 2023-07-20

**Authors:** Nfn Kiran, Meena Kashi, Shahbaz Khan

**Affiliations:** 1 Pathology and Laboratory Medicine, Staten Island University Hospital, Staten Island, USA; 2 Pathology, Staten Island University Hospital, Staten Island, USA; 3 Gastrointestinal, Hepatobiliary and Transplant Pathology, Indiana University School of Medicine, Indianapolis, USA; 4 Hematopathology, Northwell Health, New York, USA; 5 Pathology, University of Oklahoma Health Sciences Center, Oklahoma City, USA

**Keywords:** immunohistochemistry(ihc), trichome stain, helicobacter pylori, helicobacter pylori eradication, collagenous gastritis

## Abstract

*Helicobacter pylori* is the most common infection and is mostly asymptomatic in infected individuals. Only a few cases of collagenous gastritis associated with *H. pylori* infection have been reported in the previous literature. We report a case of a 54-year-old female presenting with heartburn and epigastric pain associated with bloating, gas, and sometimes constipation. The physical examination was unremarkable with a soft, non-tender, and non-distended abdomen. Upper endoscopy showed erythema in the stomach with non-erosive gastritis. Our patient was diagnosed with *H. pylori*-associated chronic active gastritis with collagenous gastritis on histologic evaluation of the gastric biopsy specimen. After treatment with *H. pylori* eradication therapy, patients with collagenous gastritis associated with *H. pylori* infections showed a significant improvement in collagenous gastritis on endoscopy.

## Introduction

An infectious disease spread through oral-oral and fecal-oral routes during adolescence, *Helicobacter pylori* can colonize gastric spaces by modulating immune responses and cause mucosal damage and chronic inflammation. *Helicobacter pylori*-induced gastritis can cause chronic gastritis with varying degrees of severity in all patients [1]. Globally, *H. pylori* infections are 50% prevalent in populations around the world. Gastritis is a common outcome of *H. pylori* infections [2].

*Helicobacter pylori* has an inner (cytoplasmic) membrane, a periplasm with peptidoglycan, and an outer membrane for its structure. The outer membrane consists of phospholipids and lipopolysaccharides (LPS) with cholesterol glucosides (a rare quality in bacterial membranes). *Helicobacter pylori* has a unique variation in outer membrane structure that allows bacteria to adapt to harsh and different gastric mucosal environments [3].

*Helicobacter pylori* can evade the host immune system by escaping Toll-like receptor (TLR) recognition, tolerating dendritic cells (DCs), blocking T-cell proliferation, inducing Treg skewing, and upregulating programmed death ligand 1 (PD-L1), and it ultimately avoids detection and persists in the host [1]. Inflammation is initiated as a protective response to infection or injury in the body. In the case of infections, damaged epithelial cells secrete damage-associated molecular patterns (DAMPs) and pathogen-associated molecular patterns (PAMPs) from *H. pylori.* Inflammation develops in response to PAMPs from *H. pylori* and DAMPs that are secreted from damaged epithelial cells. The innate immune system by interacting with pattern recognition receptors (PRRs) is activated by DAMPs, and the chronic inflammatory response is promoted by DAMPs [4].

Gastritis induced by *H. pylori* infections leads to the activation of PRRs on antigen-presenting cells (APCs), gastric epithelial cells, and inflammatory cells (neutrophils) [1]. *Helicobacter pylori* can be histologically diagnosed with hematoxylin and eosin (H&E) staining, but more specific stains such as modified Giemsa, Warthin-Starry silver, Genta, and immunohistochemical (IHC) are also used [5]. Pathologically, infected individuals have polymorphonuclear leukocyte infiltrate in the gastric mucosa and a reduction in gastric acid output [2]. According to a study, mucus on the gastric mucosa, diffuse redness, spotty redness of the fundic mucosa, enlarged fold, and mucosal edema, among others, were found to be associated with gastritis due to *H. pylori* [6]. *Helicobacter pylori* binds to connective tissue proteins and induces pro-inflammatory chemokines, leading to fibrosis and collagen deposition [7]. Coexisting *H. pylori* infection in patients with collagenous gastritis has been reported in the literature. Herein, we present a case of collagenous gastritis associated with *H. pylori* infection.

## Case presentation

**Figure 1:**
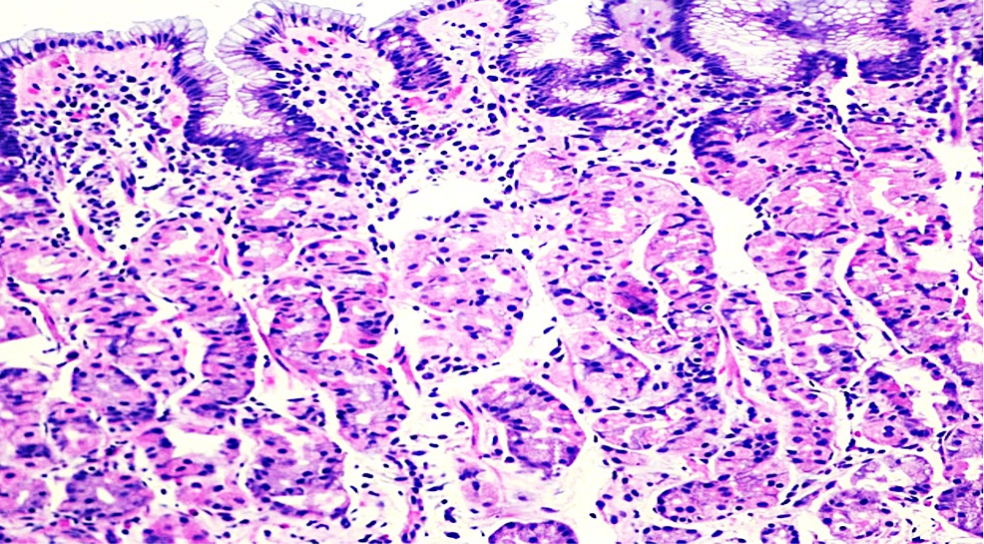
H&E stain showed chronic active gastritis associated with H. pylori and thickened collagen, 20x. H&E: hematoxylin and eosin.

**Figure 2:**
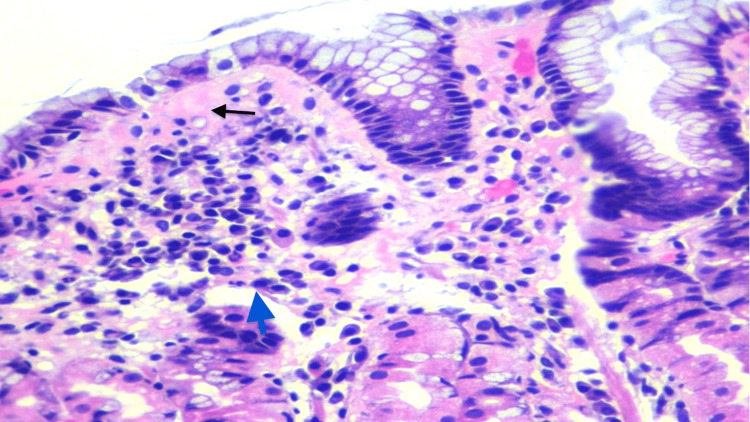
H&E stain showed chronic inflammatory cells, plasma cells (blue arrow), and thickened collagen under the gastric epithelium (black arrow), 40x. H&E: hematoxylin and eosin.

A 54-year-old female with a past medical history of diabetes mellitus (DM) presented with heartburn and epigastric pain that was sharply associated with bloating or gas, and the patient occasionally complained of constipation. There was no history of weight loss or appetite changes, dysphagia or odynophagia, coffee ground emesis, melena, and blood in feces. Physical examination was unremarkable with a soft, non-tender, and non-distended abdomen. Upper endoscopy showed esophageal hiatal hernia and erythema in the stomach with non-erosive gastritis. Colonoscopy revealed a 4 mm polyp in the sigmoid colon. Histologic evaluation of gastric biopsy showed *H. pylori*-associated chronic active gastritis with increased subepithelial collagen (Figure 1). Within and underneath the collagen table, there are scattered mixed inflammatory cells (plasma cells, eosinophils, and neutrophils) (Figure 2).

 

**Figure 3:**
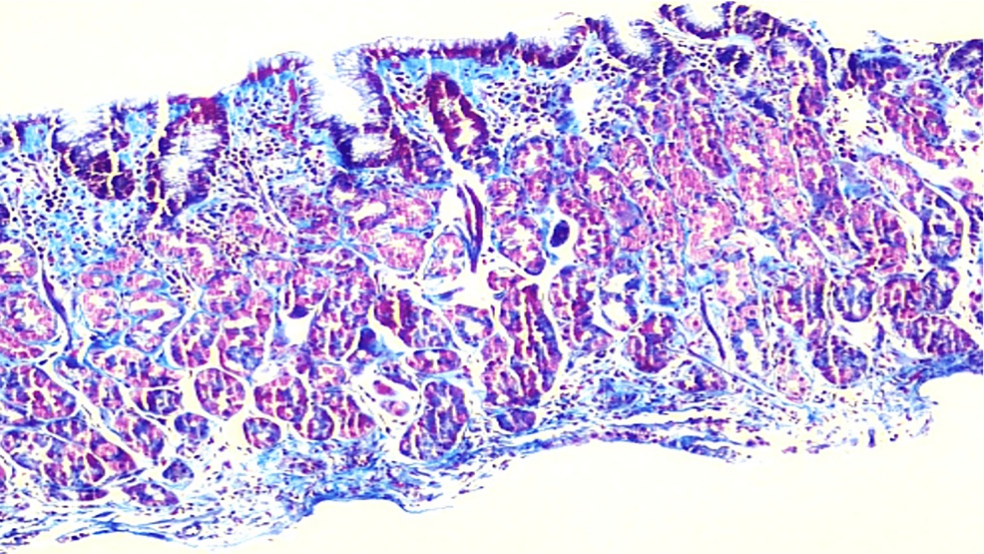
Masson trichrome stain, 40x.

Masson trichrome stain showing a subepithelial collagenous band (blue color) (Figure 3). 

**Figure 4:**
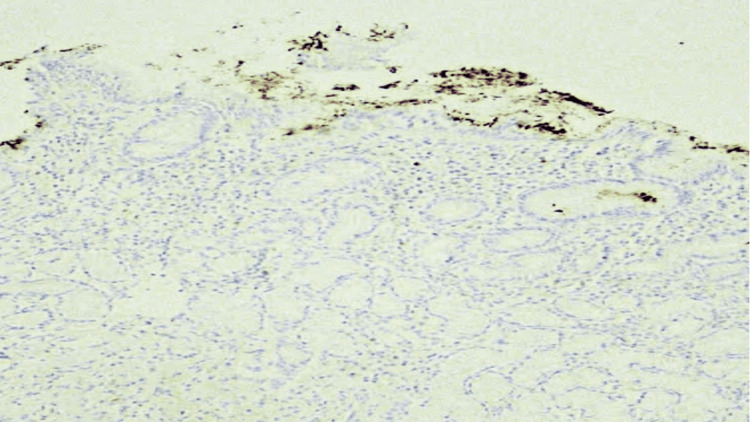
Immunohistochemistry for H. pylori showed positive staining for H. pylori, 20x.

Immunohistochemistry for H. pylori showed positive staining for *H. pylori* (Figure 4).

Based on the clinical presentation, upper gastrointestinal (GI) endoscopic findings, histopathologic analysis, and immunohistochemical evaluation, a final diagnosis of collagenous gastritis was made. 

## Discussion

The exact pathogenesis of *H. pylori* remains a mystery, but it seems that chronic inflammation due to a persistent proinflammatory environment can promote its existence. According to a study, collagen types I, II, III, and tenascin are produced due to chronic inflammation [7,8]. In clinical settings, *H. pylori* may have different pathologies due to diversity in the organism’s virulence factor production and varied response to inflammation [7]. The host responds to injury by typically activating interleukin (IL)-1b production in the gastric mucosa, which will further promote inflammatory cell (neutrophil) recruitment and activation, and upregulated IL-8 generates IL-1b [7]. 

It has been reported that at least 85% of patients with *H. pylori*-associated diseases exhibit the presence of the bacterium in the antrum, and up to 15% of patients with this organism involve the body of the stomach. In 74% of cases, *H. pylori* was found and erythematous gastritis was the commonest endoscopic finding [9]. The specificity of 92% and sensitivity of 22% with a positive predictive value of 86% to *H. pylori* infection had antral erosion [9].

Collagenous gastritis is a rare disease, first reported in 1989 by Colletti and Trainer [10]. Histologically, collagenous gastritis is characterized by the presence of thick subepithelial collagen bands, which are typically greater than 10 mm in thickness and are also associated with an inflammatory infiltrate in the gastric mucosa [10]. To date, collagenous gastritis with coexisting *H. pylori* infection has been reported in only four cases [11]. The mechanism of collagen deposition in patients who have collagenous gastritis is unknown. In collagenous gastritis, type III collagen is released to repair inflammatory damage caused by subepithelial fibroblasts [12]. Collagenous gastritis is a reparative response and not a primary pathological response [12]. *Helicobacter pylori* may not be an invasive pathogen, but when it binds to connective tissue proteins and induces pro-inflammatory chemokines, it produces extracellular matrix-like proteins, resulting in fibrosis and collagen deposition [7]. Currently, patients with collagenous gastritis associated with *H. pylori* infections are recommended a 14-day course of triple therapy of amoxicillin, proton pump inhibitor (PPI), and either clarithromycin or metronidazole [13]. Patients should be tested for eradication of *H. pylori* at least four weeks after completion of therapy [13]. The patient was clinically improved, and histologic findings were resolved when we did endoscopy. Some individuals with collagenous gastritis may experience periods of remission with appropriate treatment, while others may have persistent symptoms or recurrent flares. 

## Conclusions

Only a few cases of coexisting *H. pylori* infection and collagenous gastritis have been updated in the literature. Collagenous gastritis is diagnosed through a combination of clinical symptoms, endoscopic examination, and histopathological analysis of biopsied tissue samples. Regular follow-up of the disease with a gastroenterologist is crucial to monitor the disease progression and its management. We are reporting this case as it is a rare entity, and it will help clinicians to diagnose and manage patients with collagenous gastritis associated with *H. pylori* infection.
